# Adoptive Transfers of CD4^+^CD25^+^ Tregs Raise Foxp3 Expression and Alleviate Mouse Enteritis

**DOI:** 10.1155/2018/9064073

**Published:** 2018-09-30

**Authors:** Kai Wang, Tongjia Zhu, Haijun Wang, Jinxin Yang, Shuaishuai Du, Guoying Dong, Zhihua Pei, Guixue Hu

**Affiliations:** ^1^College of Animal Science and Technology, Jilin Agricultural University, Changchun, Jilin, China; ^2^Wildlife Ambulance Breeding Center of Jilin Province, Changchun, Jilin, China; ^3^College of Global Change and Earth System Science, Beijing Normal University, Beijing, Jilin, China

## Abstract

CD4^+^CD25^+^Foxp3^+^ Tregs control the immune response and maintain immune homeostasis. This study examined whether Tregs can affect mouse enteritis and the Foxp3 (Forkhead transcription factor) transcriptional pathway. Mouse CD4^+^CD25^+^ Treg cells were labelled using CFSE (5,6-carboxyfluorescein diacetate succinimidyl ester) and transferred to enteritis model mice. The mice were randomly divided into an enteritis group, a Treg-infusion group, a Treg-inhibiting group, and a control group. Histopathology, ELISA, flow cytometry, western blot, immunohistochemistry, and immunofluorescence were performed. Our results demonstrated that CD4^+^CD25^+^ Tregs were successfully transferred. The disease activity index (DAI) scores in the Tregs-infusion group were lower than those of the enteritis and Tregs-inhibiting groups. The number of goblet cells and inflammatory cells was reduced, and the levels of IL-1*β*, TNF-*α*, NO, and PGE2 were significantly decreased in the Tregs-infusion group compared to those in the enteritis group (p<0.05). The number of CD4^+^CD25^+^Foxp3^+^ Tregs and CD4^+^IL-17A^+^ Th17 cells in the mesenteric lymph nodes differed significantly from the enteritis and Tregs-inhibiting groups (p<0.05). There were more Foxp3^+^ Tregs and Smad3 and NFAT2 infiltrated into the duodenum after adoptive transfer of CD4^+^CD25^+^ Tregs, which was a significant difference relative to the enteritis group (p<0.05). This study demonstrated that adoptive transfer of CD4^+^CD25^+^ Tregs can decrease mouse enteritis. Foxp3 expression may be improved through the Smad3 and NFAT2 signalling pathways.

## 1. Introduction

The enteritis remains one of the leading causes of morbidity and mortality worldwide, despite ongoing progress in our basic understanding of its epidemiology, pathogenesis, and treatment [1]. The aetiology and pathogenesis of bacterial enteritis are very complicated, and they involve immunology, genetics, endocrinology, several environmental factors, and so on. These results were very important in preventing and controlling bacterial enteritis. Regulatory T cells (Tregs) are a subpopulation of T cells that can be classified into naturally occurring Tregs (nTregs) and induced Tregs (iTregs) based on their origin. CD4^+^CD25^+^ Tregs are the most common nTregs. Recent studies have demonstrated that Tregs are closely related to immunoregulation, which is involved in the body's immune stability [2], transplantation tolerance [3], tumour immunity [4], allergic reactions [5], and microorganism infection [6–9]. Reduction in the number of CD4^+^CD25^+^ Tregs, defects in surface molecule expression, and damaged parts of the immune function are associated with intestinal inflammation. Some studies from either selective deletion of Foxp3 in CD4^+^Foxp3^+^ T cells or enforced expression of Foxp3 in CD4^+^CD25^−^ T cells indicated that Foxp3 is required for programming a normal profile of Treg cells and is a reliable hallmark for Treg cells, especially in the mouse [10–12]. Therefore, Foxp3, as a transcription factor, is only expressed in mice CD4^+^CD25^+^ Treg cells and is a master regulator for the development and function of Treg cells [13].

The exact mechanism of Tregs in enteritis is not clear, but the identification of costimulatory molecules involved in the function of Tregs may facilitate further characterization of these cells [14, 15]. For example, in peripheral blood, CD4^+^CD25^+^ Tregs from IBD patients retain their suppressive activity. However, they are increased during remission and decreased during active disease [16]. Treg cells can suppress inflammation and immune responses via various mechanisms including cell-contact-dependent and independent pathway [17]. Given the above information, we propose that Treg may play a role in bacterial enteritis. In previous studies, our experimental results showed that* Lactobacillus casei* regulates differentiation of Th17/Tregs cells to reduce mouse intestinal inflammation [18]. To further study the role of CD4^+^CD25^+^ Tregs in enteritis, CD4^+^CD25^+^ Tregs were adoptively transferred through the mouse tail vein. We evaluated the anti-inflammatory effect of CD4^+^CD25^+^ Tregs and the transcriptional regulation of FoxP3 (Forkhead transcription factor 3). These results helped our understanding of the mechanism of inflammatory diseases.

## 2. Materials and Methods

### 2.1. E. coli Culture

ETEC K88 C83912 and* Lactobacillus casei* ATCC393 were provided by the Institute of Microbiology, College of Animal Science and Technology, Jilin Agricultural University.

### 2.2. Experimental Animals

SPF BALB/c male mice weighing 18~20 g were purchased from Beijing HFK Bioscience CO., LTD (Beijing, China). The mice were housed in polystyrene cages with stainless steel wire lids and given water and chow ad libitum. The housing was maintained at a constant temperature (21°C-22°C) with a 12-hour light-dark cycle. The mice were utilized at 4-6 weeks of age. The protocol was approved by the Committee on the Ethics of Animal Experiments of the Jilin Agricultural University. The mice were euthanized according to the laboratory animal management regulations of China.

### 2.3. Experimental Design

A mouse enteritis model was produced as previously described [19]. Enteritis group: the mice were pretreated with 0.2 ml 1% NaHCO_3_. After 30 min, 1.0 × 10^8^ CFU/ml ETEC K88 was administered. Treg-infusion group: CFSE-labelled CD4^+^CD25^+^Foxp3^+^ Tregs (1.0 × 10^6^ cells/mouse) were adoptively transferred to enteritis mice using tail vein injection. Treg-inhibiting group: Cyclosporine A (CsA)+0.9% NaCl was injected into the abdominal cavity (CsA 20 *μ*g/g weight) [20].

### 2.4. Purification of Treg and Adoptive Cell Transfer

Cells from the mesenteric lymph nodes were analyzed for CD4^+^CD25^+^Foxp3^+^Treg expression using a Treg cell staining reagent according to the manufacturer's instructions. Briefly, CD4^+^ cells from mice were enriched using positive selection with anti-CD4 microbeads and MACS. Next, we used positive selection with anti-CD25 microbeads and MACS according to the instructions provided by the manufacturer (BD Biosciences, USA). The absolute number of cells was calculated from the data obtained from the Attune flow cytometer and correlated with the total cell number counted by the trypan blue exclusion test. We used a 1.0 × 10^6^ dose to investigate the effect of CD4^+^CD25^+^Foxp3^+^ Tregs on inflammation as previously reported [21]. Tregs were injected intravenously in the tail in a 200 *μ*l suspension.

### 2.5. Measurement of Cytokine Levels

The concentrations of IL-1*β*, TNF-*α*, NO, and PGE2 in sera were determined using ELISA kits (Lengton Biotech Co., Ltd. China) according to the manufacturer′s protocols.

### 2.6. Western Blot Analysis

Total protein was drawn from mesenteric glands using a total protein extraction sample kit (Nanjing Keygen Biotech Co., Ltd. China). Protein quantification was performed using the BCA protein quantification kit (Nanjing Keygen Biotech Co., Ltd. China). The protein samples were resolved using SDS-PAGE. After electrophoresis, proteins were transferred and blocked in 5% skim milk (overnight at 4°C). The appropriate amounts of rat anti-Smad3, NFAT2, and Stat3 mAb (Abcam, USK) were added and blocked in the shaker for 2.5 hours at 37°C. The membranes were washed three times with 1×TBST. Goat anti-mouse IgG (Abcam, USK) was added. The membrane protein was posted on the X-ray film for exposure and developed in the developing machine. The signal intensity was analysed using grey-scale analysis software (ImageTool 3.0).

### 2.7. Histopathologic Examination

Tissue samples were collected and set in 10% buffered formalin solution, processed and embedded in paraffin for histopathological analysis. Three mm thick sections were stained with haematoxylin and eosin (H&E). Inflammatory changes were evaluated in 5 sections from each sample. Three samples from each experimental group from 3 different experiments were analysed.

### 2.8. Immunofluorescent Analysis

Duodenum samples were fixed with 4% paraformaldehyde and embedded in paraffin. Paraffin sections (4-5 *μ*m) were deparaffinized, rehydrated, and underwent antigen retrieval. The sections were incubated with rabbit anti-mouse CD4 (Abcam) and rat anti-mouse Foxp3 (eBioscience) antibodies after blocking. Secondary antibodies, FITC-labelled goat anti-rat Ab for Foxp3, and Cy3 goat anti-rabbit Ab for CD4 were used. The signal was detected using the Olympus Provis fluorescence microscope (Nikon Eclipse Ti-SR) with 200× magnification. The mean fluorescence intensity (MFI) was calculated using Image-Pro Plus 6.0.

### 2.9. Immunohistochemical Analysis

Duodena were collected from each experimental group after 72 h transfer, fixed with 4% paraformaldehyde, and embedded in paraffin. Paraffin sections (4-5 *μ*m) were deparaffinized, rehydrated, and treated with PBS. Antigens were retrieved. After blocking for 10 min with milk seal, the sections were incubated with anti-Smad3∖NFAT2∖Stat3 (Abcam) antibodies overnight at 4°C and treated using PBS. A rabbit-specific HRP/DAB (ABC) Detection IHC Kit (Abcam) was used with a haematoxylin and eosin counterstain. The average optical density (AOD) was calculated using Image-Pro Plus 6.0.

### 2.10. Statistical Analyses

The results are presented as the means ± SD from six mice per group. The statistical analysis was performed using the SPSS 18.0 software program. Differences between the groups were compared using one-way ANOVA, and the differences were considered statistically significant at P<0.05.

## 3. Results

### 3.1. CD4^+^CD25^+^ Tregs Can Be Transferred into the Mouse Body

**Figure 1:**
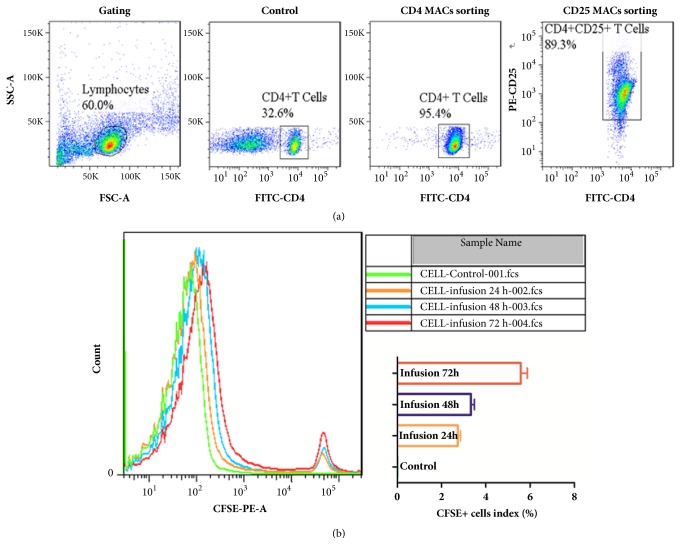
**CD4** ^+^ **CD25** ^+^ **Tregs by labelled CFSE were successfully transferred into the mouse body**. (a) The percentage of CD4^+^CD25^+^ Tregs was 89.3%. (b) CD4^+^CD25^+^ Tregs labelled with CFSE can be detected after adoptive transfer at 24 h, 48 h, and 72 h. Six mice per group were used in the study.

CD4^+^CD25^+^ Treg cells from mice were first purified using MACS, and they were 89.3% pure (Figure 1(a)). To analyse whether the transferred Tregs can be successfully transferred into the mouse body, CD4^+^CD25^+^ Tregs were labelled using CFSE and transferred to healthy mice. The percentage of CD4^+^CD25^+^ Tregs was tested after adoptive transfer at 24 h, 48 h, and 72 h (Figure 1(b)), and the results showed that CD4^+^CD25^+^ Tregs had be transferred into the mouse body.

### 3.2. Adoptive Transfer of Tregs Reduces an Inflammatory Response in ETEC K88-Infected Mice

**Figure 2:**
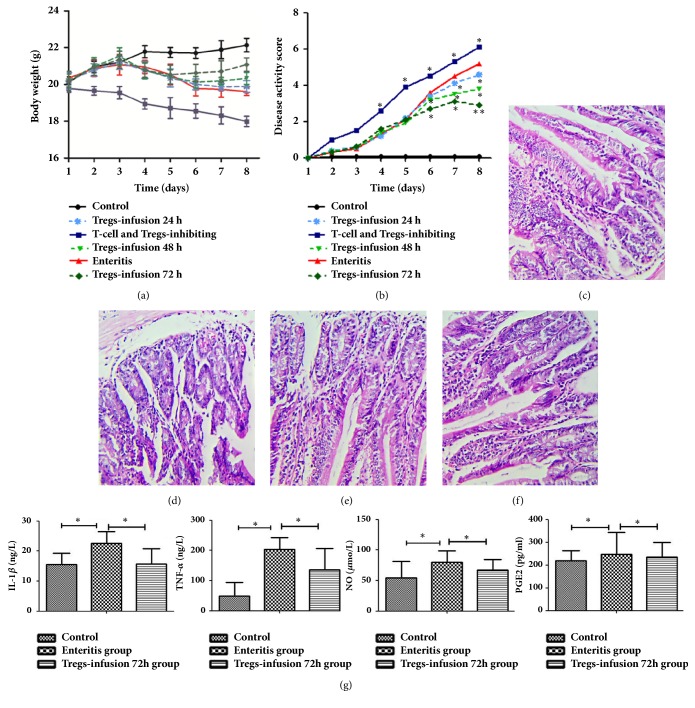
**Adoptive transfer of Tregs induces an inflammatory response in ETEC K88-infected mice**. Mice infected with ETEC K88, either with transferred Tregs or not, were euthanized. (a) Weight change. (b) Disease activity score (DAS). (c-f) Duodenum was obtained and histological studies were carried out using H&E staining (200×). Representative images from mice that were (c) uninfected, (d) from the enteritis group, (e) after transfer at 72 h, and (f) from the Treg-inhibiting group. Representative data from 3 independent experiments is shown. Six mice per group were used in the study. (g) The levels of IL-1*β*, TNF-*α*, NO, and PGE2 in the peripheral blood. *∗*p<0.05 and *∗∗*p<0.01 compared with the corresponding enteritis group, one-way ANOVA.

To analyse whether the increased Tregs number was related to the inflammatory reaction, we carried out adoptive transfer of Tregs in mice. Mice in the Tregs-inhibiting group started to lose weight on the first day. However, mice in the Treg-infusion group showed gradual weight gain (Figure 2(a)). The DAI scores of the Treg-inhibiting group were higher than those of the other groups, and that of the enteritis group was higher than that of the Treg-infusion group starting at the fifth day (Figure 2(b)). To analyse the change in inflammation, we analysed morphological changes in the duodenum after transfer at 72 h. Uninfected control mice with transferred Tregs showed no change in the duodenum (Figure 2(c)). Enteritis mice showed severe destruction of the intestinal mucosa. Infiltration of inflammatory cells, such as lymphocytes and neutrophils, was observed in the lamina propria and mucous layers (Figure 2(d)). Tregs-infusion mice showed a decreased inflammatory infiltrate, mainly in mucosa and intestinal glands, and the number of goblet cells and inflammatory cells was reduced (Figure 2(e)). The changes in Tregs-inhibiting mice were similar to those observed in the Tregs-infusion group (Figure 2(f)). To study whether the reduction of duodenal inflammation was a consequence of the Treg transfer response, we measured the levels of inflammatory factors in the peripheral blood using ELISA. The levels of IL-1*β*, TNF-*α*, NO and PGE2 were decreased compared to those in the Treg-transferred mice and were significantly different compared to the levels in the enteritis group (P<0.05) (Figure 2(g)). Uninfected control mice with transferred Tregs were also studied using the same methods, but we did not detect a statistically significant result.

### 3.3. Affecting the Th17/Tregs Differentiation by Adoptive Transfer of Treg Cells

**Figure 3:**
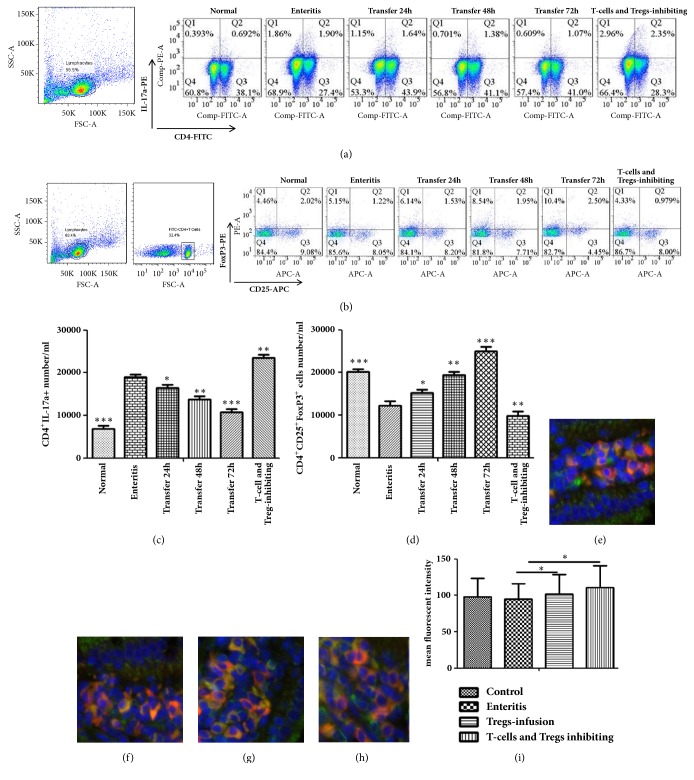
**Upsetting the Th17/Tregs balance using adoptive transfer of Tregs**. At 72 h after enteritis, Tregs were transferred to mice, and the mesenteric lymph node was obtained. (a-d) Representative FACS analysis of intracellular detection of Th17 and Tregs in the mesenteric lymph node. Ten thousand events from both subgates were captured. Normal: normal group, Enteritis: enteritis group, Transfer 24 h: after transfer 24 h, Transfer 48 h: after transfer 48 h, Transfer 72 h: after transfer 72 h, and T-cells and Tregs-inhibiting: T-cells and Tregs-inhibiting group. (e-i) Representative immunofluorescence results of CD4^+^Foxp3^+^ on CD4 T cells in the duodenum from different groups of mice (200×) and mean fluorescent intensity (MFI) were analysed, (e) uninfected group, (f) enteritis group, (g) Treg-infusion group, and (h) T-cells and Treg-inhibiting group. Data are the means ± SD from 3-8 mice/group; Foxp3+ cells are green, CD4^+^ cells are red, and CD4^+^Foxp3^+^ Treg cells are yellow. Results represent 1 experiment repeated 3 times. Six mice per group were used in the study. *∗*p < 0.05; *∗∗*P < 0.01 or *∗∗∗*P < 0.001, one-way ANOVA.

Th17 and Treg cells in the mesenteric lymph node were measured using flow cytometry. The percentage of Th17 cells in the Treg-infusion group was measured. It was highly expressed in the enteritis group, and this resulted in a significant difference (p<0.05). It was obviously expressed in the Tregs-inhibiting group (p<0.05) (Figures 3(a) and 3(c)). According to the result of Treg analysis, Tregs were obviously expressed in the Tregs-infusion group and poorly expressed in the enteritis group, and the difference was significant (p<0.05). However, Tregs were poorly expressed in the Tregs-inhibiting group (Figures 3(b) and 3(d)). Next, we measured the levels of CD4^+^CD25^+^ Tregs in different groups after transfer at 72 h. The Foxp3 protein content increased significantly, and its content was higher than that of the enteritis group (p<0.05). The level of Foxp3 expression significantly increased (p<0.05) compared to the enteritis group (Figures 3(f), 3(g), and 3(h)). This result showed that the dynamic expression of Th17 and Treg cells was closely associated with the occurrence and outcome of intestinal inflammation.

### 3.4. Foxp3 Expression Was Improved via the Smad3 and NFAT2 Signalling Pathways

**Figure 4:**
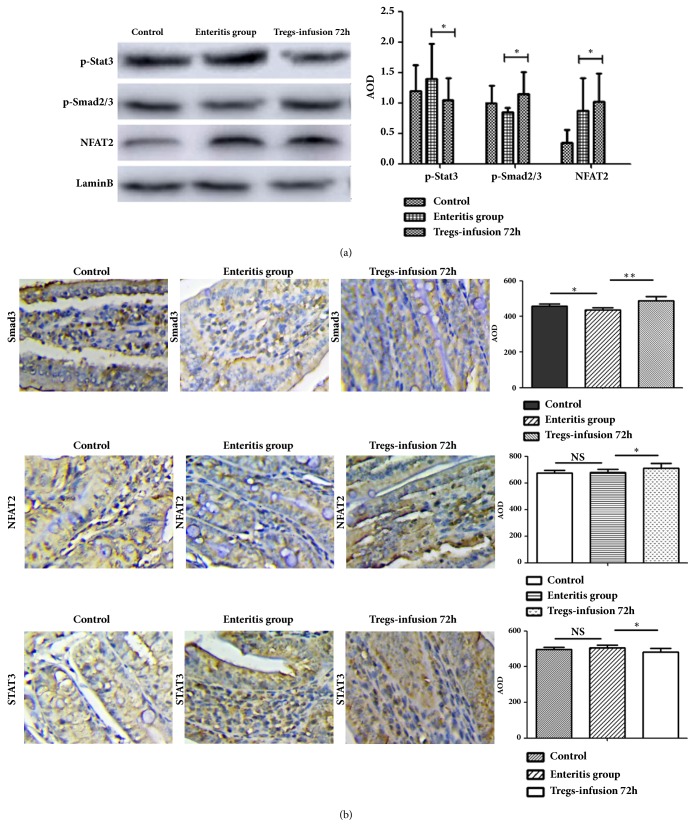
**Expression of Smad3, NFAT2, and Stat3 in the duodenum**. (a) The results of Smad3, NFAT2, and Stat3 western blot analysis. (b) The average optical density (AOD) of Smad3, NFAT2, and Stat3 in the duodenum was analysed using immunohistochemistry. The sections are represented as a percentage of the total duodenum (200×). Results represent 1 experiment repeated 3 times. Six mice per group were used in the study. *∗*P < 0.05 and NS: not significant.

To study FoxP3 gene transcriptional control, after transfer at 72 h, the Smad3 (Drosophila mothers against decapentaplegic 3), NFAT2 (Nuclear factor of activated T cells 2), and Stat3 (signal transducer and activator 3) proteins were detected using western blot. The results showed that the Smad3 and NFAT2 proteins were obviously increased after adoptive transfer (p<0.05), and Stat3 was obviously decreased (p<0.05) (Figure 4(a)). In the duodenum, the expression of Smad3, NFAT2, and Smad3 was measured using immunofluorescence. The results showed that there was more Smad3 and NFAT2 infiltrate in the duodenum after the transfer of Tregs (p<0.05, Figure 4(b)). After transfer at 72 h, the proportion of Stat3 in the Tregs-infusion group decreased to the control level (p<0.05, Figure 4(b)). These results demonstrated that Smad3 and NFAT2 play an important role in Foxp3 transcription.

## 4. Discussion

CFSE (5,6-carboxyfluorescein diacetate succinimidyl ester) fluorescent markers have been successfully applied in cell proliferation, cell toxic effect, and cell tracking studies. To study CD4^+^CD25^+^ Tregs proliferation in mice, CD4^+^CD25^+^ Tregs in mouse mesenteric lymph nodes were separated using immune magnetic beads. The results showed that the proportion of CD4^+^CD25^+^ Tregs was 89.3%. The CD4^+^CD25^+^ Tregs were labelled using CFSE adoptive transfer to mice through the tail vein. After transfer at 24 h, 48 h, and 72 h, CD4^+^CD25^+^ Tregs were successfully transferred into the mouse body.

After adoptive transfer, the weight of the transfer mouse group increased gradually, the DAI reduced, and duodenal pathological histology inspection found that the intestinal goblet cells and inflammatory cells decreased. IL-1*α* and TNF-*α* are proinflammatory cytokines secreted by helper T cells, dendritic cells, and other cells during inflammation, and they can induce the occurrence and expansion of inflammation. NO and PGE2 are inflammatory mediators. In this study, the levels of IL-1*α*, TNF-*α*, NO, and PGE2 in the peripheral blood of the Treg-infusion group were significantly lower. These results demonstrated that adoptive transfer of Tregs can reduce the mouse intestinal inflammatory reaction.

Th17 cells are proinflammatory cells that secrete inflammatory factors such as IL-17a. ROR*γ*t (retinoid-related orphan receptor-*γ*) is a specific transcriptional regulator required for Th17 cells differentiation. Tregs function in the negative regulation of the immune system, and they play a critical role in immune self-tolerance. Tregs express FoxP3 [22], which is important in the development and outcome of various diseases, including cancer, infectious diseases, and transplantation immunity [23, 24]. Tregs and Th17 cells have been described as two distinct subsets and have the opposite effects on autoimmunity [25]. In our previous study, we revealed that a Th17/Tregs imbalance exists in mice with ETEC-induced intestinal inflammation. In this study, we investigated whether the Th17/Tregs functional imbalance existed after CD4^+^CD25^+^ Tregs were adoptively transferred. Th17 cells numbers were significantly lower, and the CD4^+^CD25^+^Foxp3^+^ Treg cells numbers were significantly higher. This result demonstrated that the differentiation of Th17 and Treg cells was regulated after adoptive transfer. CsA is a kind of immunosuppressant, and previous studies showed that Treg can be effectively inhibited in peripheral blood [20]. After transfer at 72 h, the number of CD4^+^CD25^+^ Tregs obviously increased and the difference was significant. This result showed that the transfer of CD4^+^CD25^+^Foxp3^+^ Tregs plays an important role.

Foxp3 is a master regulator in the development and function of Treg cells [10–12]. Stable Foxp3 expression does not act as an on/offswitch [26, 27], but it is a prerequisite for the maintenance of the transcriptional and functional programme established during Tregs development. However, Foxp3 expression is regulated at the molecular level by focusing on factors, such as NFAT, Smad, and STAT. Some studies showed that FoxP3 expression is controlled by transcription factors. For example, Smad3 binds to intronic enhancer 1 of the Foxp3 locus -85 bp upstream of the transcriptional start site [28, 29]. NFAT2 binding to the enhancer region substantially increased at later times, and it was responsible for maintaining Foxp3 expression [30, 31]. In this study, the expression of Smad3 and NFAT2 in the Treg-infusion group was significantly higher than that in the enteritis group, but the expression of Stat3 was lower than that in the enteritis group. This result showed that Smad3 and NFAT2 have a positive impact on the regulation of Foxp3 transcription.

A limitation of the present study is the need for further confirmation of the results; for example, treatment with Smad3 and NFAT2 inhibitors in the cell culture supernatant and knocking down the expression of certain genes (such as Smad3 and NFAT2) could provide further support for our results. Despite this limitation, the present data significantly contribute to the understanding of the underlying anti-inflammatory mechanisms of Adoptive transfer of CD4^+^CD25^+^ Tregs.

## 5. Conclusions

This study suggests that the transfer of CD4^+^CD25^+^ Tregs can stabilize Foxp3 gene expression, which can influence the differentiation of Th17/Tregs, and mouse enteritis was reduced.

## Data Availability

The data used to support the findings of this study are available from the corresponding author upon request.

## References

[1] Steiner T. S., Samie A., Guerrant R. L. (2006). Infectious diarrhea: New pathogens and new challenges in developed and developing areas. *Clinical Infectious Diseases*.

[2] Sakaguchi S., Wing K., Onishi Y., Prieto-Martin P., Yamaguchi T. (2009). Regulatory T cells: how do they suppress immune responses?. *International Immunology*.

[3] Todo S., Yamashita K. (2018). Anti-donor regulatory T cell therapy in liver transplantation. *Human Immunology*.

[4] Mougiakakos D., Choudhury A., Lladser A., Kiessling R., Johansson C. C. (2010). Regulatory T cells in cancer. *Advances in Cancer Research*.

[5] Palomares O., Yaman G., Azkur A. K., Akkoc T., Akdis M., Akdis C. A. (2010). Role of Treg in immune regulation of allergic diseases. *European Journal of Immunology*.

[6] Long E., Wood K. J. (2009). Regulatory T cells in transplantation: Transferring mouse studies to the clinic. *Transplantation*.

[7] Vila J., Isaacs J. D., Anderson A. E. (2009). Regulatory T cells and autoimmunity. *Current Opinion in Hematology*.

[8] Joosten S. A., Ottenhoff T. H. M. (2008). Human CD4 and CD8 regulatory T cells in infectious diseases and vaccination. *Human Immunology*.

[9] Scully P., MacSharry J., O'Mahony D. (2013). Bifidobacterium infantis suppression of Peyer's patch MIP-1*α* and MIP-1*β* secretion during Salmonella infection correlates with increased local CD4+CD25+ T cell numbers. *Cellular Immunology*.

[10] Khattri R., Cox T., Yasayko S., Ramsdell F. (2003). An essential role for Scurfin in CD4+CD25+ T regulatory cells. *Nature Immunology*.

[11] Fontenot J. D., Gavin M. A., Rudensky A. Y. (2003). Foxp3 programs the development and function of CD4+CD25+ regulatory T cells. *Nature Immunology*.

[12] Hori S., Nomura T., Sakaguchi S. (2003). Control of regulatory T cell development by the transcription factor Foxp3. *Science*.

[13] Sakaguchi S. (2005). Naturally arising Foxp3-expressing CD25^+^CD4^+^ regulatory T cells in immunological tolerance to self and non-self. *Nature Immunology*.

[14] Huang C.-T., Workman C. J., Flies D. (2004). Role of LAG-3 in regulatory T cells. *Immunity*.

[15] Read S., Malmström V., Powrie F. (2000). Cytotoxic T lymphocyte-associated antigen 4 plays an essential role in the function of CD25^+^ CD4^+^ regulatory cells that control intestinal inflammation. *The Journal of Experimental Medicine*.

[16] Maul J., Loddenkemper C., Mundt P. (2005). Peripheral and intestinal regulatory CD4+CD25^high^ T cells in inflammatory bowel disease. *Gastroenterology*.

[17] Wan Y. Y., Flavell R. A. (2008). TGF-*β* and regulatory T cell in immunity and autoimmunity. *Journal of Clinical Immunology*.

[18] Wang K., Dong H., Qi Y. (2017). Lactobacillus casei regulates differentiation of Th17/Treg cells to reduce mouse intestinal inflammation. *Canadian Journal of Veterinary Research*.

[19] Wang K., Qi Y., Yi S., Pei Z., Pan N., Hu G. (2016). Mouse duodenum as a model of inflammation induced by enterotoxigenic Escherichia coli K88. *Journal of Veterinary Research*.

[20] Scottà C., Esposito M., Fazekasova H. (2013). Differential effects of rapamycin and retinoic acid on expansion, stability and suppressive qualities of human CD4+CD25+FOXP3+ T regulatory cell subpopulations. *Haematologica*.

[21] Feng M., Wang Q., Jiang Z. (2016). Adoptive transferred hepatic stellate cells attenuated drug-induced liver injury by modulating the rate of regulatory T cells/T helper 17 cells. *Clinical Immunology*.

[22] Ruggieri S., Frassanito M. A., Dammacco R., Guerriero S. (2012). T reg lymphocytes in autoimmune uveitis. *Ocular Immunology and Inflammation*.

[23] Waight J. D., Takai S., Marelli B. (2015). Cutting edge: Epigenetic regulation of Foxp3 defines a stable population of CD4+ regulatory T cells in tumors from mice and humans. *The Journal of Immunology*.

[24] Maggio R., Viscomi C., Andreozzi P. (2014). Normocaloric low cholesterol diet modulates Th17/Treg balance in patients with chronic hepatitis C virus infection. *PLoS ONE*.

[25] Zhou L., Lopes J. E., Chong M. M. W. (2008). TGF-Β-induced Foxp3 inhibits TH17 cell differentiation by antagonizing ROR*γ*t function. *Nature*.

[26] Nagar M., Vernitsky H., Cohen Y. (2008). Epigenetic inheritance of DNA methylation limits activation-induced expression of FOXP3 in conventional human CD25-CD4+ T cells. *International Immunology*.

[27] Polansky J. K., Kretschmer K., Freyer J. (2008). DNA methylation controls Foxp3 gene expression. *European Journal of Immunology*.

[28] Tone Y., Furuuchi K., Kojima Y., Tykocinski M. L., Greene M. I., Tone M. (2008). Smad3 and NFAT cooperate to induce Foxp3 expression through its enhancer. *Nature Immunology*.

[29] Samon J. B., Champhekar A., Minter L. M. (2008). Notchi and TGF 21 cooperatively regulate Foxp3 expression and the maintenance of peripheral regulatory T cells. *Blood*.

[30] Mantel P., Ouaked N., Rückert B. (2006). Molecular mechanisms underlying FOXP3 induction in human T cells. *The Journal of Immunology*.

[31] Wang H., Zhao L., Sun Z., Sun L., Zhang B., Zhao Y. (2006). A potential side effect of cyclosporin A: Inhibition of CD4+CD25+ regulatory T cells in mice. *Transplantation*.

